# Exacerbation of AMD Phenotype in Lasered CNV Murine Model by Dysbiotic Oral Pathogens

**DOI:** 10.3390/antiox10020309

**Published:** 2021-02-18

**Authors:** Pachiappan Arjunan, Radhika Swaminathan, Jessie Yuan, Mohamed Elashiry, Amany Tawfik, Mohamed Al-Shabrawey, Pamela M. Martin, Thangaraju Muthusamy, Christopher W. Cutler

**Affiliations:** 1 Department of Periodontics, Dental College of Georgia, Augusta, GA 30912, USA; rswaminathan@augusta.edu (R.S.); jeyuan@augusta.edu (J.Y.); moelashiry@augusta.edu (M.E.); chcutler@augusta.edu (C.W.C.); 2 Vision Discovery Institute, Augusta University, Augusta, GA 30912, USA; amtawfik@augusta.edu (A.T.); malshabrawey@augusta.edu (M.A.-S.); pmmartin@augusta.edu (P.M.M.); 3 Department of Oral Biology and Diagnostic Sciences, Augusta University, Augusta, GA 30912, USA; 4 Department of Biochemistry & Molecular Biology, Augusta University, Augusta, GA 30912, USA; mthangaraju@augusta.edu

**Keywords:** age-related macular degeneration, retinal inflammation, oxidative stress, antioxidants, retinal degeneration, periodontal disease, therapeutic targets

## Abstract

Emerging evidence underscores an association between age-related macular degeneration (AMD) and periodontal disease (PD), yet the biological basis of this linkage and the specific role of oral dysbiosis caused by PD in AMD pathophysiology remains unclear. Furthermore, a simple reproducible model that emulates characteristics of both AMD and PD has been lacking. Hence, we established a novel AMD+PD murine model to decipher the potential role of oral infection (ligature-enhanced) with the keystone periodontal pathogen *Porphyromonas gingivalis*, in the progression of neovasculogenesis in a laser-induced choroidal-neovascularization (Li-CNV) mouse retina. By a combination of fundus photography, optical coherence tomography, and fluorescein angiography, we documented inflammatory drusen-like lesions, reduced retinal thickness, and increased vascular leakage in AMD+PD mice retinae. H&E further confirmed a significant reduction of retinal thickness and subretinal drusen-like deposits. Immunofluorescence microscopy revealed significant induction of choroidal/retinal vasculogenesis in AMD+PD mice. qPCR identified increased expression of oxidative-stress, angiogenesis, pro-inflammatory mediators, whereas antioxidants and anti-inflammatory genes in AMD+PD mice retinae were notably decreased. Through qPCR, we detected *Pg* and its fimbrial 16s-RrNA gene expression in the AMD+PD mice retinae. To sum-up, this is the first in vivo study signifying a role of periodontal infection in augmentation of AMD phenotype, with the aid of a pioneering AMD+PD murine model established in our laboratory.

## 1. Introduction

Nearly 11 million Americans have age-related macular degeneration (AMD) [1,2,3,4,5]. The National Eye Institute (NEI) reports that this number is expected to double by 2050, and 1 in 20 people with early AMD is susceptible to having advanced AMD 10 years later [6]. Worldwide, AMD is the third leading cause of blindness imposing high healthcare and economic burden [3,7,8,9]. AMD is a late-onset neurodegenerative disease which chiefly targets a small central area of light-sensitive cells in the retina responsible for sharp vision in humans. The early stage of AMD is characterized by drusen deposits, the hallmark of AMD, between the retinal pigment epithelial (RPE) cell layers and the Bruch’s membrane. The late stage is presented as dry form and/or the formation of choroidal neovascularization (CNV), which represents the wet or neovascular form (nAMD) [10,11]. Although, palliative treatment is available for “wet” form of AMD, no proven therapy is available for the “dry” type. Major risk factors for AMD include advanced age, family history, smoking, high blood pressure, and being Caucasian [6,12]. Smoking and body mass index are regarded as the modifiable risk factors for progression to nAMD, characterized by uncontrolled growth of new leaky blood vessels under the macula. However, genetics and other environmental risk factors play a crucial role in the pathogenesis of AMD [13]. Whilst the specific etiology of AMD still remains inexplicit, a growing body of evidence implicates the involvement of low-grade chronic inflammation and altered immune components, oxidative stress, mitochondrial dysfunction, and abnormal lipid metabolism [14,15,16,17,18,19]. Furthermore, an infectious etiology is now implicated in the onset and progression of AMD [20,21,22,23,24]. The specific microbiota of human nasopharynx, intestinal, and intraocular microbiota have been identified to mediate the pathogenesis of AMD [21,22,23,24]. A high-antibody titer of gram-negative bacteria like *Chlamydia pneumoniae* [25] and *Helicobacter pylori* [26] is reported in dry and wet AMD patients [27]. Nonetheless, the sequelae of molecular or cellular events that result in AMD are sparsely understood [28]. Owing to an escalating prevalence, complex multifactorial etiology, and lack of effective cure, acquirement of a better understanding of the pathophysiological mechanisms is indispensable for revealing new therapeutic targets.

Emerging studies imply periodontitis or periodontal disease (PD) as a risk factor in the development of AMD [29,30,31,32,33,34]. PD is a microbially-induced chronic inflammatory disease that affects both the soft and hard tooth-supporting tissues leading to tooth loss. PD is a world-wide disease of epidemic proportions, with prevalence in the US reaching nearly 50% [35]. PD has a bidirectional relationship with a myriad of systemic disorders ranging from rheumatoid arthritis to Alzheimer’s disease [35,36,37]. The latest experimental observations from our group and others suggest an association between PD and retinal degenerative diseases, particularly AMD [20,28,38,39]. Chronic oral inflammation sustained by dysbiosis of the “proximal gut”, i.e., the oral cavity and a leaky attachment apparatus, induces aberrant systemic immune responses directly through the dissemination of periodontal pathogens or indirectly via the microbial biproducts into the blood circulation from the damaged periodontal pockets [28,40]. The periodontal pathogens are not confined to local inflammation but also impact serious systemic disorders of high morbidity and mortality [35,36,37,41,42]. In the pathogenesis of many systemic diseases including PD and AMD, the role of oxidative stress and antioxidants is significantly remarked. Several scientific studies have addressed the crosstalk between oxidative stress and inflammation in AMD pathophysiology. The “oxidation-specific epitopes” generated in response to pathological oxidative damage induce pro-inflammatory responses and promoted macrophage infiltration and polarization [43,44]. Furthermore, oxidative stress contributes to inflammation and angiogenesis and in turn, retinal neurodegeneration as in the case of AMD. On the other hand, the periodontal pathogens activate neutrophils which release the reactive oxygen species (ROS) and cause destruction of the periodontal tissues [45]. Our and other studies with mouse models have shown that lack of antioxidant genes have demonstrated AMD phenotypes such as subretinal deposits, RPE atrophy, and neovascularization [46,47]. Antioxidants are useful elements in the management of AMD as they are found to attenuate the progression of early AMD to advanced stages. In this context, a positive correlation between antioxidant levels with attenuation of PD and periodontal wound healing has been speculated [48]. A cross-sectional study by Chapple et al. indicates that the total antioxidant capacity was significantly reduced in both gingival crevicular fluid (GCF) and plasma of patients with PD relative to healthy controls [49]. AMD shares many common risk factors with PD which modify their onset and progression [12,27,50], but precise knowledge of the role of PD in the pathophysiology of AMD is currently lacking.

*Porphyromonas gingivalis* (*P. gingivalis/Pg*) the primary pathogen in PD, is a dysbiotic species renowned for its ability to influence the host directly by tissue infiltration and damage and indirectly cause distal inflammation by hijacking the immune system through its metabolic biproducts to support its survival and multiplication [28,51,52]. It expresses an assortment of virulence factors that contribute to its pathogenic effects both locally and systemically [53]. The primary factors include fimbriae, hemagglutinin, Arg- and Lys-gingipains that degrade Ig and various components of complement system, and the outer-membrane vesicles which aid in colonization and attachment. Also expressed by *Pg* is a polysaccharide capsule and a unique lipopolysaccharide (LPS). These are typically involved in modulation of host responses, host tissue damage, spreading infection, and fibrinolytic, keratinolytic, and other hydrolytic activities [54,55]. From those listed, gingipains are major virulence factors which exist as lysine-gingipain (Kgp), arginine-gingipain-A(RgpA) and arginine-gingipain-B (RgpB) [38,56]. PD promote entry of bacteria into the blood stream, which activates the host inflammatory response through several cascade mechanisms [57]. *P. gingivalis* binds to and invades epithelial cells [58,59,60], dendritic cells (DCs) [61], and macrophages through diverse strategies [62]. Numerous animal studies have reported that infection with *P. gingivalis* that impairs endothelial integrity, accelerates atherosclerosis, and causes a significant increase in mortality due to cardiac rupture. The production of high levels of nitric oxide [63], pro-inflammatory cytokines, cell adhesion molecules [64], and chemokines by the infected endothelial cells have important effects on the vascular function [65]. Farrugia et al. showed that the gingipains present on the outer-membrane vesicles surface mediate the vascular events leading to increased vascular permeability, involving endothelial cell–cell adhesins [66]. Following systemic dissemination, *P. gingivalis* initiates several processes involved in atherosclerotic heart disease, including platelet aggregation and leukocyte extravasation to subendothelial regions [66,67]. Moreover, *P. gingivalis* were abundantly retrieved from healthy vascular tissue of atherosclerotic patients [68].

We have previously confirmed a distinct two-hit process by which *P. gingivalis* using its *Mfa1*(short) and *FimA*(long) fimbriae, induces DC-specific intercellular adhesion molecule-3-grabbing non-integrin-mediated immunosuppression, and promotes chemokine-receptor type4-mediated pathological angiogenesis [39]. Recently, we reported invasion and intracellular survival of *Pg* in the RPEs in vitro [20]. However, there is no in vivo study available to clarify the potential association between the pathogenesis of retinal degenerative diseases, particularly AMD and PD. The purpose of the current pilot study is to investigate the role of *P. gingivalis* in the pathophysiology of retinal degeneration and pre-existing AMD phenotype. Herein, we established a unique AMD+PD murine model and observed aggravation of AMD phenotypes including vasculogenesis, drusenoid deposits with activation of inflammatory mediators, oxidative stress, and inactivation of key antioxidants molecules. This is the first report to demonstrate the involvement of *Pg* and its biofilm consortium members in the augmentation of AMD pathology, specifically neovascular changes and retinal degeneration in AMD+PD mice retinae.

## 2. Materials and Methods

### 2.1. Bacterial Strains and Biofilm Culture

Wild-type *Porphyromonas gingivalis*-381 (*Pg*), *Fusobacterium nucleatum* (ATCC49256; *Fn*), and *Streptococcus gordonii DL1 (Sg*) [69] were routinely cultured, and the growth curve was established as described previously [38,39]. Briefly, both *Pg* and *Fn* were cultured anaerobically (10% H_2_, 10% CO_2_, and 80% N_2_) in Difco^TM^ Anaerobe Broth MIC (BD Biosciences San Jose, CA) in a Forma Scientific anaerobic system glove box, while *Sg* established in aerobic condition at 37 °C. Bacterial suspensions were washed three times in PBS and re-suspended for spectrophotometer reading of 0.11 for optical density at 660 nm, which was previously determined to be equal to 5 × 10^7^ colony-forming units (CFU) [70]. To prepare the polybacterial (biofilm) suspension for infection, the initial colonizer *S. gordonii* was mixed with an equal quantity of *F. nucleatum* (intermediate colonizer) for 5 min; subsequently, *P. gingivalis* (late colonizer) were added to the culture tube, and cells were mixed thoroughly and allowed to interact for an additional 5 min. *P. gingivalis* or biofilm (*Sg+Fn+Pg*) suspension were then mixed with an equal volume of sterile 2% carboxymethylcellulose (CMC; Sigma-Aldrich, St. Louis, MO, USA) in phosphate-buffered saline (PBS). This mixture was used for periodontal infection (1 × 10^9^ CFU) in C57BL/6J mice as described [12,17] below.

### 2.2. Animals

**Figure 1:**
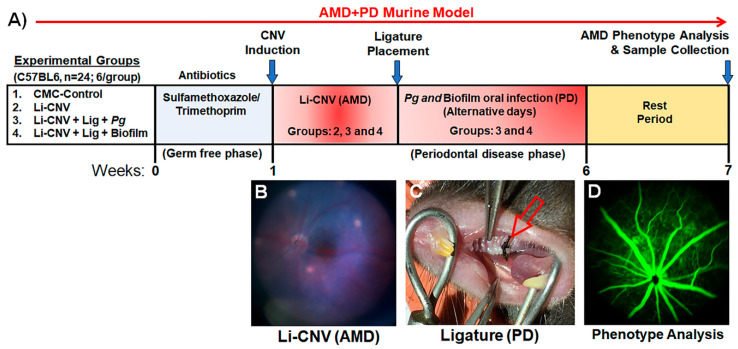
Study design and establishment of unique age-related macular degeneration (AMD)+ periodontal disease (PD) murine model. (**A**) Shows the complete experimental design involving four groups 1. CMC (−^ve^) control, 2. Laser-induced choroidal neovascularization (Li-CNV), 3. Li-CNV+Lig+Pg, and 4. Li-CNV+Lig+Biofilm (detailed in the methods section). (**B**,**C**) Establishment of unique AMD+PD murine model. After laser induction, mice were ligated followed by *Pg* or biofilm infection and AMD+PD model established. Representative image demonstrating the laser induction (**B**), ligature placement around the second maxillary molar (**C**-red arrow), and systematic noninvasive fundus fluorescein angiography (FFA) (**D**) for AMD phenotype analysis.

Wild-type C57BL/6J mice were purchased from the Jackson Laboratory (Bar Harbor, ME). All mouse experimental procedures described in this study have been reviewed and approved by the Augusta University Institutional Animal Care and Use Committee (AUP#: 2017-0836), in compliance with established federal and state policies. Mice were maintained in individually ventilated cages and fed a normal chow diet. Mice were treated with a week regimen of oral antibiotics (sulfamethoxazole/trimethoprim suspension 48 mg/mL) to allow for *P. gingivalis* or biofilm-specific colonization. Mice were used for experiments at the age of 10 weeks and were divided into four groups (1. CMC (−^ve^) control, 2. Laser-induced choroidal neovascularization (Li-CNV; +^ve^) control, 3. Li-CNV+ligature+*Pg*, and 4. Li-CNV+Ligature+biofilm) of six mice each (Figure 1). Two groups had their left maxillary second molar tooth ligated, and two groups were left un-ligated as described below.

### 2.3. Laser-Induced Choroidal Neovascularization (Li-CNV) AMD Model

The formation of laser-induced CNV in a mouse model simulates the exudative (neovascular or wet) AMD in humans, so the Li-CNV model was established as we described previously [71]. Briefly, 10-week-old male mice were anesthetized, and their were pupils dilated after a week of antibiotics treatment. Mice were positioned on a rack connected to a slit lamp delivery system. Four laser photocoagulation spots were made (75 µm spot size, 75 ms, 90 mW power, Oculight Infrared Laser System 810 nm, IRIDEX Corporation) in the area surrounding the optic nerve in the right eye of mice (left-intact non-CNV control). The sites were visualized through a handheld contact lens and a viscous surface lubricant. Only laser-induced burns with a bubble formation were included in the study. The mice were given lubricant ophthalmic ointment after laser treatment. Ligature induction were performed as described below after laser treatment. CNV area was analyzed after 1 week.

### 2.4. Ligature-Enhanced Periodontitis (PD) in Pre-Existing CNV (AMD) Model

To test the influences of *Pg* or biofilm consortium members in pathological neovascularization, we established the ligated periodontitis model [72,73] with the Li-CNV (wet AMD) mouse as described above, 3 days post laser treatment. This model emulates the human AMD+PD. The animals underwent general anesthesia with a combination of ketamine/xylazine (80 mg/kg + 5 mg/kg body weight, respectively) administered via intraperitoneal injection. The ligature model was obtained by placing a sterilized 5–0 silk thread (Roboz Surgical Instrument Co., MD, USA) around the maxillary upper-left second molar and tied mesiobuccally for groups 3. Li-CNV+Lig+*Pg* and 4. Li-CNV+Lig+Bio to compare to the untreated groups (1. CMC control, 2. Li-CNV control—no ligature or bacteria). Suture was applied and tied gently to prevent damage to the periodontal tissue. The ligatures remained in place in all mice throughout the experimental period without the necessity of replacement. Mice were challenged by oral application of vehicle (2% CMC in PBS, no bacteria) or the *P. gingivalis* alone and biofilm (1 × 10^9^ CFU) at the buccal surface of the maxillary vestibule for 6 weeks on alternative days with 1 week of rest before study completion. Mice were analyzed noninvasively by fundus photography, Optical coherence tomography, and fluorescein angiography at 1 and 6 weeks as described below. After 6 weeks of chronic infection, mice were sacrificed at the 7th week, and the eyes were removed for RNA isolation, H&E, and immunostaining analysis (Figure 1).

### 2.5. Fundus-Imaging Analysis

One and six weeks after the oral infection course, fundus images were obtained in all groups to compare the CNV lesion and chorio-retinal vascular changes. In brief, each mouse received an intramuscular injection of rodent anesthesia cocktail (ketamine 80 mg/kg, xylazine 5 mg/kg) in 0.1–0.2 mL of phosphate-buffered saline (PBS). Next the pupils were dilated with 1.0% tropicamide (Alcon, Fort Worth, TX, USA), and their corneas locally anesthetized with 0.5% proparacaine (Alcon). A retinal-imaging microscope system (Micron IV, Phoenix Research Labs, Pleasanton, CA, USA) was utilized for color fundus photography, Fundus Fluorescein angiography (FFA), and image-guided optical coherence tomography (OCT).

### 2.6. Optical Coherence Tomography (OCT)Analysis

To assess the presence of angiogenesis, imaging of eyes was performed after 1 and 6 weeks using OCT imaging device with a 25-diopter lens fitted on a 30-degree angle lens. The pupils of the anesthetized mice were dilated with 1% tropicamide eye drops before the images were acquired. A lubricant eye gel (GenTeal; Novartis Pharmaceuticals, East Hanover, NJ, USA) was used throughout the procedure to maintain moisture and clarity in the cornea. Three repeated-volume intensity projections were acquired for each eye. The images consisted of 50–100 averaged B-scans.

### 2.7. Fundus Fluorescein Angiography (FFA) Analysis

To evaluate the retinal vasculature and permeability in vivo, one and six weeks after oral gavage/infection with *Pg* and biofilm, mice were anesthetized using intramuscular injection of the rodent anesthesia cocktail described above [74]. The pupils were dilated with 1% tropicamide eye drops, and the mice were placed on the imaging platform of the Phoenix Micron III retinal-imaging microscope (Phoenix Research Laboratories, Pleasanton, CA, USA). Goniovisc 2.5% (hypromellose; Sigma Pharmaceuticals, LLC, Monticello, IA, USA) was applied generously to maintain moisture in the eyes during imaging. Mice were administered 10 to 20 μL 10% fluorescein sodium (Apollo Ophthalmics, Newport Beach, CA, USA), and rapid acquisition of fluorescent images ensued for ∼5 min. Fluorescein leakage manifests as indistinct vascular borders progressing to diffusely hazy fluorescence. Fluorescein leakage (CNV area) was compared between different groups by quantifying the fluorescence intensities collected after 1, 2, and 3 min following fluorescein injection using ImageJ software (National Institutes of Health, Bethesda, MD, USA).

### 2.8. Analysis of Retinal Thickness

Retinal-thickness analysis was performed as we described previously [47]. Briefly, the eyes of *Pg-* and biofilm-infected and control mice were harvested after 6 weeks. To ensure that the same locations of the eyes were analyzed and compared, a mark was placed at the 12:00 o’clock position of the corneal limbus of each eye. The eyes were then embedded in optimal-cutting-temperature compound (Crystalgen Inc., Commack, NY, USA). Ten micrometer frozen sections were cut in parallel to the 12:00 o’clock meridian through the optic nerve and fixed in 4% paraformaldehyde and H&E stained. Retinal layer thickness was measured in a blinded manner regarding the genotypes at six locations of each eye: 25% (S1), 50% (S2), and 75% (S3) of the distance between the superior pole and the optic nerve, and 25% (I1), 50% (I2), and 75% (I3) of the distance between the inferior pole and the optic nerve. Only the sections with the optic nerve visible were used for analysis. The slides which contain 6 eye sections per slide from different groups were scanned. Digital slide scanning was carried out using the Leica Aperio Versa and images analyzed by Aperio ImageScope software from Leica (Microsystems Inc., Buffalo Grove, IL, USA). The thickness of the ONL and IS/OS retinal layers was measured at 200× magnification using Leica software and image analyzer. The similar procedures were used for the noninvasive images taken from optical coherence tomography.

### 2.9. Immunofluorescence and Confocal-Imaging Analysis

Immunofluorescence analysis was performed using optimal-cutting-temperature compound embedded-eye-tissues sections as we described previously [20,39]. In brief, after fixing and permeabilizing, the tissues from mice retinae were blocked with 1X universal power block (Biogenex Laboratories, Fremont, CA, USA) and incubated for 30 min at room temperature. After blocking with permeabilization buffer and 2% BSA, slides were then incubated with rabbit polyclonal collagen-IV alpha 1 primary antibody followed by antirabbit FITC-conjugated secondary antibody (Novus Biologicals, Littleton, CO, USA). After washing with PBS twice, subsequently, slides were mounted with prolong diamond antifading mounting medium with the nuclear probe DAPI (Invitrogen, Carlsbad, CA, USA). Images were acquired with a Zeiss LSM510 inverted meta scanning confocal microscope. Specificity of the reaction was confirmed by omitting the primary antibody. For quantitative analysis, images were collected from six sections per mouse of at least three mice in each group and then analyzed using ImageJ software.

### 2.10. Quantitative PCR Assays

Total RNA was isolated as we described [38,39] using TRIzol reagent (Waltham, MA, USA) and purified with RNeasy kit (Germantown, MD, USA) from orally infected with *Pg* alone, biofilm, and uninfected control mice retinae. RNA quantity and integrity were tested and only ratios of absorbance at 260 and 280 nm of 1.8–2.0 were included in this study. Analysis of gene expression in mice retinae orally infected by *Pg* and biofilm were performed using RT-PCR as we described previously [39]. Complementary DNA was synthesized from 1.0 μg RNA through a reverse-transcription reaction (Applied Biosystems, Foster City, CA, USA). Quantitative PCR was performed on TaqMan^®^ gene-expression primers and probes for fast plates in triplicates for selected genes: Tnf-α (Assay ID: Mm00443258_m1), Tgf-β1 (Assay ID: Mm1178820_m1), Il-1β (Assay ID: Mm00434228_m1), Il-6 (Assay ID: Mm00446190_m1), Il-10 (Assay ID: Mm01288386_m1), Foxp3 (Assay ID: Mm00475162_m1), 18S-rRNA (Assay ID: Mm03928990_g1), and Gapdh (Assay ID: Mm99999915_g1). Gene expression of specific bacterial strains including *Pg381* (Assay ID: AIY9ZZ2), *Mfa-1* (Assay ID: AIX01UM), and *FimA* (Assay ID: AIY9ZZQ) were quantified using universal 16S-rRNA (Assay ID: BQ04230899_s1) as housekeeping. qPCR was performed as well as reconfirmed with SYBR green methods for selected angiogenesis, oxidative stress, and antioxidant genes (PCR primers are listed in Table S1). For each biological sample, three experimental replicates were analyzed and repeated thrice. Fold regulations were calculated using the 2^−ΔΔCt^ method as we described previously [38]; β-actin, Gapdh, and 18S-rRNA were used as internal controls.

### 2.11. Statistical Analysis

Statistical analyses were carried out using the GraphPad Prism 8.4.3 software (GraphPad, San Diego, CA, USA). The analysis of variance was performed by one-way ANOVA and multiple comparisons using the Dunnett’s and/or Tukey’s test. Two groups were compared to an unpaired Student’s t test and two-tail p value. All data were reported as the mean ± standard error of the mean (SEM) obtained from at least three independent experiments. P values less than 0.05 were considered statistically significant.

## 3. Results

### 3.1. Establishment of Experimentally Induced AMD+PD Murine Model

There are several independent PD [75] and AMD [76,77] animal models available which emulate the “natural” pathogenesis of each of these human diseases. Thus far, a simple reproducible model that incorporates the features of both AMD+PD in a single model is presently unavailable. We established a laser-induced choroidal neovascularization (Li-CNV) mouse model as shown in the study design (Figure 1A,B) [71,78,79]. The Li-CNV mice were then ligated (Figure 1C) and orally infected with *Pg* alone and with biofilm as illustrated (Figure 1A and methods section). We utilized this combined model to test our hypothesis that chronic oral infection with *P. gingivalis* or biofilm exacerbates pathological neovascularization and retinal degeneration in pre-existing AMD. This unique reproducible model mimics human AMD+PD as shown in Figure 1B,C. This experimentally induced animal model is a useful tool to study the influence of oral pathogens such as *P. gingivalis* (in mono- and polymicrobial infections) in the pathogenesis of AMD (Figure 1D). We emphasize here that this is the first report of a combined AMD+PD pioneer model established to study the impact of PD in pre-existing AMD.

### 3.2. Increased Vascular Leakage and CNV Area in AMD+PD Mice Retinae.

**Figure 2:**
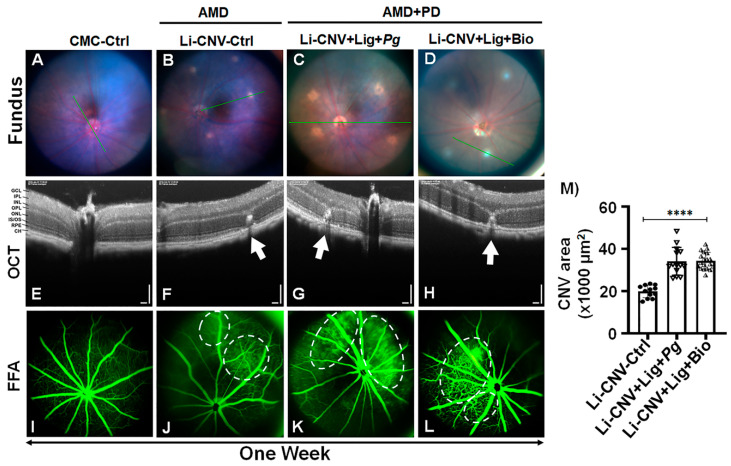
Increased retinal vascular leakage by oral pathogens in AMD+PD mice retinae. (**A**–**D**) Representative fundus photography from mice before and after laser in a subsequent week. Fundus show (**A**) CMC-control group (2% CMC, no bacteria) and spots of laser burn (**B**) Li-CNV control, (**C**) Li-CNV+Ligature+*Pg*, and (**D**) Li-CNV+Lig+Biofilm after 1 week (n=24). The green lines indicate the scan lines of SD-OCT sections. (**E**–**H**) Representative images of SD-OCT and (**I**–**L**) fundus fluorescein angiography (FFA) after 1 week of CMC-control, Li-CNV-Control, Li-CNV+Lig+*Pg*, and Li-CNV+Lig+Biofilm-infected mice retinae, respectively. (**F**–**H**) OCT shows rupture of Bruch’s membrane (BM, white arrows) induced by laser burn compared to CMC-control. Refer the low-magnification image for the complete view (Figure S1). Representative FFA images after injection of fluorescent dye 1 week post laser burn (**J**–**L**) and CMC-control (**I**). FFA demonstrates increased blood leakage (white circle) in *Pg* (**K**) and biofilm (**L**) infected after 1-week compared to Li-CNV-control (**J**). Refer Figure S1 for another set of representative FFA images. (**M**) Quantification analysis shows increased CNV areas in *Pg*- and biofilm-infected mice retinae after 1 week compared to CNV control. Mean ± SEM analyzed by one-way ANOVA followed by Dunnett’s multiple comparisons test (*n* = 18, **** *p* < 0.0001).

The noninvasive fundus image showed laser-burn spots in Li-CNV, Li-CNV+Ligature+*Pg*, and Li-CNV+ Ligature+ Biofilm-infected groups compared to CMC control (Figure 2 A–D) at one week. The spectral domain optical coherence tomography (SD-OCT) further confirmed the rupture of Bruch’s membrane induced by laser burn compared to CMC (−^ve^)-control (Figure 2E–H; Figure S1D,H,L). We also observed reduced retinal thickness in biofilm-infected mice retinae compared to CNV and CMC control (Figure 2H; Figure S1K,L). Next, we analyzed induction of vascular leakage in AMD+PD mice retinae by fundus fluorescein angiography (FFA). The FFA found pronounced blood leakage in both ligature-enhanced *P. gingivalis* infection (alone) and biofilm-infected Li-CNV mice retinae at 1 week compared to CNV and CMC controls (Figure 2I–L). The quantification analysis of CNV areas showed significant increase in AMD+PD compared to CNV control retinae at one week (Figure 2M). This data connotes that PD induces vascular modifications even within a short duration of one week.

### 3.3. Exacerbation of Pathological Angiogenesis and Inflammation in AMD+PD Mice Retinae

**Figure 3:**
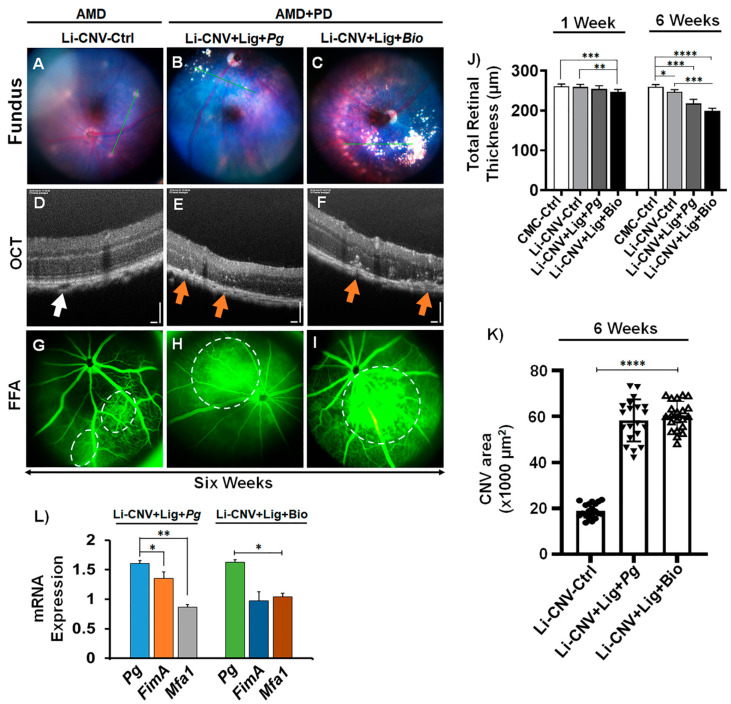
Progression of AMD pathogenesis through oral pathogens in AMD+PD mice retinae. Representative images of fundus (**A**–**C**), SD-OCT (**D**–**F**), and FFA (**G**–**I**) after 6 weeks of Li-CNV-Ctrl (**A**,**D**,**G**), Li-CNV+Lig+*Pg* (**B**,**E**,**H**) and Li-CNV+Lig+Bio (**C**,**F**,**I**) infected mice retinae, respectively. (**A**–**C**) Fundus shows increased chronic CNV lesions and drusen-like deposits in chronic *Pg*- (**B**) and biofilm (**C**)-infected retinae compared to CNV-control (A). Refer to Figure S2 for another set of representative fundus images. The green lines indicate the scan lines for the OCT sections. (**D**–**F**) OCT images further confirm chronic CNV lesion and drusenoid deposits located above the RPE in *Pg*- (**E**) and biofilm (**F**)-infected retinae after 6 weeks with obvious reduction in retinal thickness and predominant vitreal (refer to Figure S2H,M, blue arrow) and subretinal angiogenesis compared to CNV control (**D**, white arrow). Representative OCT scans show multiple subretinal and cuticular drusen-like depositions (orange arrows). Refer to the low magnification in Figure S2 for a complete view. (**G**–**I**) FFA shows increased blood leakage (dotted white circle) after 6 weeks compared to CNV and 1 week (Figure 1J–L). Refer to Figure S2 for another set of representative FFA images. (**J**) Quantification of total retinal thickness in *Pg-* and biofilm-infected mice at 1 and 6 weeks, compared to CMC and CNV controls. Total retinal thickness measured from the nerve fiber layer to the RPE layer. Mean ± SEM analyzed by one-way ANOVA followed by Tukey’s multiple comparisons test (*n* = 24; **** *p* < 0.0001; *** *p* < 0.001; and ** *p* < 0.01). (**K**) Quantification analyses show increased CNV areas in *Pg-* and biofilm-infected mice retinae after 6 weeks compared to CNV control. Mean ±SEM analyzed by one-way ANOVA followed by Dunnett’s multiple comparisons test (*n* = 18, **** *p* < 0.0001). (**L**) qPCR shows mRNA expression of *Pg*, *FimA*, and *Mfa1* in *Pg* alone and biofilm orally infected mice retinae and untreated controls, normalized with the internal (universal 16S-rRNA) control and compared to *Pg* vs. *Mfa1* and *FimA* genes (*n* = 12; ** *p* < 0.01).

Subsequently, to test the exacerbation of AMD, the mice were orally infected with *Pg* alone and with biofilm consistently for 6 weeks (chronic infection) on alternate days. Fundus showed an expanding laser burn with inflammatory lesion-like morphology or drusen-like deposits in both *Pg*- and biofilm-infected AMD+PD retinae after 6 weeks compared to CNV control (Figure 3A–C; Figure S2A–E*).* OCT imaging found apparent drusen-like deposit in the subretinal area and obvious decrease of retinal thickness in both *Pg*- and biofilm-infected Li-CNV mice at 6 weeks (Figure 3E,F; Figure S2G–J,L–O). We also observed chronic CNV lesions in *Pg* and biofilm after 6-weeks with vitreal and subretinal angiogenesis, compared to CNV control (Figure S2H,M). Next, we analyzed the vascular leakage in AMD+PD mice retinae by FFA. The results show increased blood leakage at 6 weeks in time in both *Pg*- and biofilm-infected mice retinae compared to CNV control (Figure 3G–I; Figure S2P–T). Interesting to note is that both *Pg*- and biofilm-infected mouse revealed large hyperfluorescent progressive lesions (Figure 3H,I; Figure S2Q–T) persistent after 6 weeks, relative to CNV-control (Figure 3G; Figure S2P). The quantification analysis further confirmed significant and consistent increase of CNV areas in AMD+PD mice retinae at 6 weeks compared to CNV-control (Figure 3K). These results suggest advancement of laser injury and choroidal neovascularization, a hallmark pathology of advanced wet AMD resulting from chronic PD.

### 3.4. Pg 16S-rRNA Gene Expression in the Retinae of AMD+PD Murine Model

Next, we analyzed mouse retinae for the presence of *Pg* and their virulent fimbrial gene expression in the orally infected (*Pg* and biofilm) mice retinae compared to CNV control. Here, we show the expression of *Pg* and its fimbrial genes namely *Mfa1* (short or major fimbria) and *FimA* (long or minor fimbria) in the retinae of AMD+PD murine model. We analyzed the mRNA of the retinae from AMD+PD and CNV control mice to determine *Pg* and its fimbrial gene expression as they are critical in colonization and binding to other biofilm species [51]. qPCR analysis using *Pg, FimA, and Mfa1* 16S-rRNA primers revealed *Pg* and its fimbrial 16S-rRNA gene in the mice retinae, relative to CNV/CMC and negative control, to rule-out PCR artifact in the *Pg, FimA*, and *Mfa1* PCR results. Surprisingly, *Pg*, *FimA* and *Mfa1* fimbrial genes were detected, while *Pg* were expressed significantly more than 1.5-fold in both *Pg*-alone and biofilm infected mice retinae compared to *Mfa1* and *FimA* (Figure 3L). We also observed nondetectable range of the cycle threshold of these bacterial strains and *Pg*-fimbrial mRNA expression in majority of the controls. This suggests a role of *Pg* in the *in-vivo* retinal environment, where it adapts and alter the AMD-key genes and the course of vasculogenesis.

### 3.5. Reduced Retinal Thickness in AMD+PD Mice

**Figure 4:**
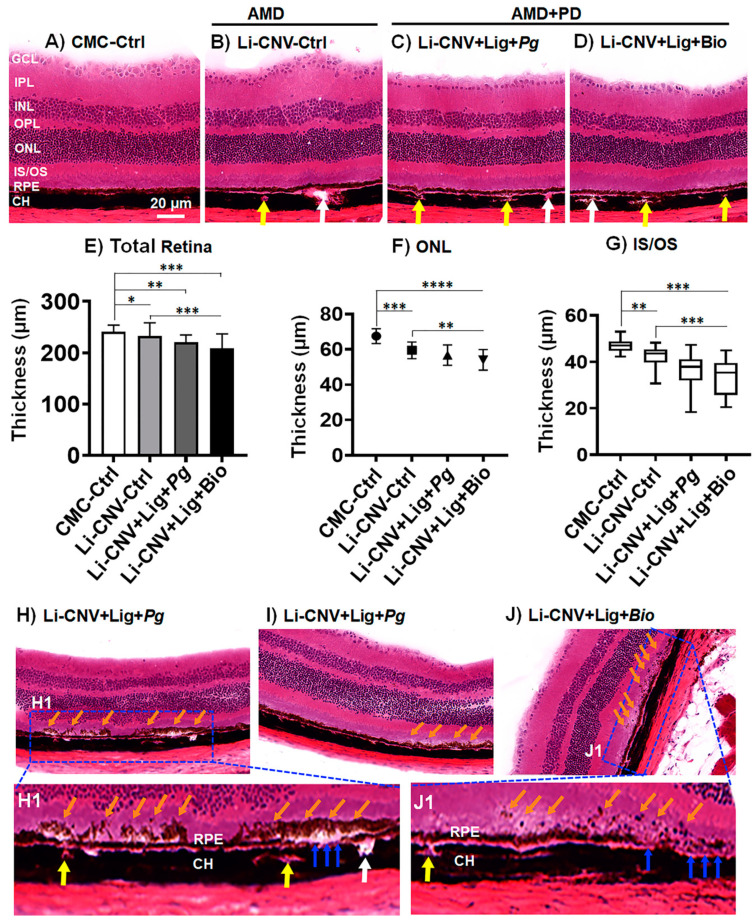
Decreased retinal thickness and formation of subretinal drusen-like deposits in AMD+PD mice. (**A**–**D**) H&E staining shows total retinal thickness of Li-CNC+Lig+*Pg* and Li-CNV+Lig+Biofilm orally infected mice model after 6 weeks compared to CMC and Li-CNV controls. (Scale bar: 20 µm). (**B**–**D**) White arrows indicate the CNV spots (empty space) while yellow arrows show the infiltration of immune cells in choriocapillaris of the AMD+PD retinae. Refer Figure S3 for another set of representative images. (**E**–**G**) Quantification analysis shows that the thickness of the retinal layers of AMD+PD mice was significantly reduced, including the outer nuclear layer (ONL), inner segment/outer segment (IS/OS). Total retinal thickness measured from the nerve fiber layer to the RPE layer. Mean ± SEM analyzed by One-way ANOVA followed by Dunnett’s and Tukey’s multiple comparisons test (*n* = 12; **** *p* < 0.0001, *** *p* < 0.001, ** *p* < 0.01, * *p* < 0.05). (**H**–**J**) Representative H&E images show drusen-like deposits in the subretinal area (orange arrows) and disintegrated RPE layer in *Pg* and Biofilm infected mice. Boxed areas in H and J show an enlarged region as H1 and J1 clearly demonstrate the RPE damage (yellow arrows) and drusenoid deposits in the subretinal area (orange arrows), respectively.

Laser-induced CNV causes retinal degeneration in mouse models [77]. However, it remains unknown whether PD plays a role in the aggravation of the retinal degeneration in pre-existing AMD animal models. To explore this, we utilized the AMD+PD mice model and investigated the morphology of the retinae by OCT imaging and quantified by ImageScope analyzer for 1 and 6 weeks. We found that, at the first week of infection, the thickness of the retinae of Li-CNV+Lig+Biofilm was significantly reduced compared to CMC and CNV controls (Figure 2J; 1 week). While at 6 weeks of chronic infection, the thickness of the retinae of both *Pg* and biofilm were significantly reduced (Figure 2J; 6 weeks). There was also a significant progressive decrease, falling to 99/94, and 94/76 % of controls at 1, and 6 weeks, respectively (Figure 2J). To substantiate the reproducibility of the retinal thickness reduction in AMD+PD mice, we further analyzed the H&E retinal sections after 6 weeks at the completion of the experiments. H&E sections showed significant reduction in retinal thickness in AMD+PD mice compared to CMC and CNV control (Figure 4A–E; Figure S3A–D). In addition, we also observed morphological variations including thickening of Bruch’s membrane and abnormal alteration of RPE layer in *Pg* and biofilm infected mice retinae (Figure 4C,D; Figure S3C,D). Thinning was observed in nearly all the retinal layers, including inner segment/outer segment (IS/OS), outer nuclear layers (ONL) of *Pg* and biofilm infected were significantly reduced (Figure 4F,G). This result was consistent with the noninvasive OCT imaging analysis (Figure 2J; 6 weeks). These data suggest that infection with oral pathogens can exacerbate the retinal degeneration in pre-existing AMD mice.

### 3.6. Subretinal Drusen-like Deposits in AMD+PD Mice

Next, we analyzed the H&E section since we observed drusenoid deposits located above the RPE in the fundus and OCT images (Figure 3 and Figure S2). Interestingly, the histology demonstrated disintegrated RPE layer with ensuing damage and drusen-like deposits in the subretinal region of the AMD+PD mice retinae (Figure 4H–J) after 6 weeks. This data was consistent with the OCT imaging analysis (Figure 3E,F and Figure S2G–J,L–O). In addition, we also observed infiltration of immune cells in the choriocapillaris and CNV area (Figure 4(H1,J1), and Figure S3(C1,D1)). This data signifies that low-grade chronic infection by the oral pathogens induce the formation of drusen-like deposits which could mimic the human drusenoid lesion and might promote RPE damage and retinal degeneration gradually. However, further study is warranted to confirm this phenomenal morphology.

### 3.7. Increased Choroidal/Retinal Neovasculogenesis in the Retinae of AMD+PD Mice

**Figure 5:**
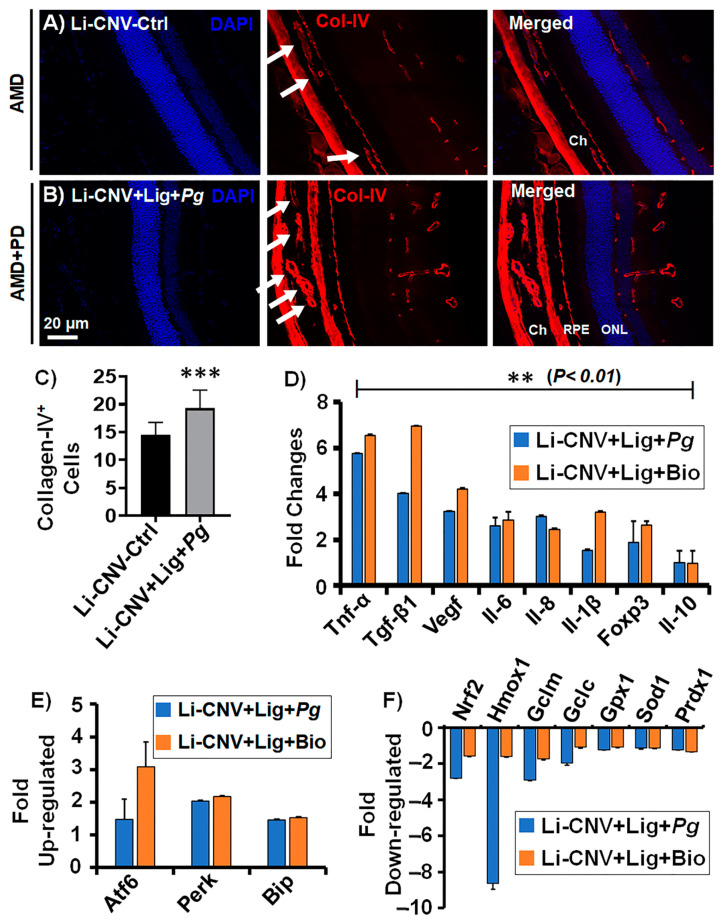
Increased choroidal/retinal vasculogenesis, oxidative stress, inflammatory mediators, and decreased antioxidants genes in the retinae of AMD+PD mice. (**A**–**C**) Immunofluorescence staining shows more Col-IV (angiogenic/vascular marker) in the AMD+PD mice retinae and choroidal region compared to CNV control (**A**). (**B**) white arrows indicate newly formed blood vessels. Note: Refer to Figure S4 for another set of representative images. (**C**) Quantification analysis shows increased Col-IV^+^ cells in Li-CNV+Lig+*Pg*-infected retinae, relative to Li-CNV group. Two groups were compared, and data were analyzed using unpaired Student’s t test (*n* = 6; *** *p* < 0.001). (Scale bar: 20 µm). (**D**–**F**) qPCR shows up-regulation of pro-inflammatory (**D**), oxidative stress (**E**) genes and down-regulation of anti-inflammatory mediators (**D**) and antioxidants (**F**) in Li-CNV+Lig+*Pg,* and Li-CNV+Lig+Bio relative to the average of CNV and CMC controls. Fold change in gene expression normalized with internal controls, and ±1.5 fold was considered significant (*n* = 6, ** *p* < 0.01).

The inference of aggravated angiogenesis and inflammatory lesions evidenced by FFA and OCT results directed us to analyze the expression level of vasculogenic marker collagen-IV (Col-IV) in the AMD+PD mice retinae. Immunofluorescence analysis demonstrated increased Col-IV^+^ cells, which are common indicators of blood vessel presence in the AMD+PD mice retinae and choroidal regions compared to CNV control (Figure 5A,B; Figure S4(1–3)). Quantitative analysis showed significantly increased vasculogenesis in AMD+PD compared to CNV control (Figure 5C). This result was consistent with the FFA imaging analysis (Figure 2M and Figure 3K). We also observed increased fibrovascular formation in the AMD+PD mice retinae by FFA (Figure S1E,F). These data collectively suggest that *Pg* promotes nAMD phenotype through inflammatory angiogenesis.

### 3.8. Increased Angiogenesis, Pro-Inflammatory and Decreased Anti-Inflammatory Mediators in AMD+PD Mice Retinae

The earlier results prompted us to test the specific angiogenic, pro- and anti-inflammatory mediators relevant to both PD and AMD conditions in AMD+PD mice retinae. qPCR analysis reveals significant (*p* < 0.01) up-regulation of angiogenic (VEGF, IL-6 and IL-8) [80], pro-inflammatory (Tnf-α, Tgf-β1, and Il-1β) [18,19] and immunosuppressive (Foxp3) genes, while there was down-regulation of the anti-inflammatory (IL-10) gene [81] in *Pg*- and biofilm-infected Li-CNV+ ligated mice retinae (Figure 5D), consistent with advanced AMD. These results indicate that persistent insult with *Pg,* and chronic over-activation leads to exacerbation of AMD phenotype by inflammatory mediators.

### 3.9. Up-Regulated Expression of Oxidative-Stress Genes and Down-Regulated Antioxidant Genes in AMD+PD Mice Retinae

Since PD is associated with oxidative stress levels and antioxidants dysfunction [45], we subsequently evaluated the mRNA expression of a group of oxidative stress and antioxidant genes in AMD+PD mice retinae. Using real-time PCR assays, we found that *Pg* and biofilm markedly up-regulated many oxidative stress genes, namely Atf6, Perk, and Bip [82,83,84,85] (Figure 5E). *P. gingivalis* and biofilm down-regulated significantly (*p* < 0.01) numerous critical antioxidative genes in the retinae of lasered ligature-induced mice, including Nrf2 [86,87], Hmox1 (Ho-1), Gclc, Gclm [88], Gpx1, Sod1, and Prdx1 [47] (Figure 5F). This data insists that *Pg* and biofilm can act as critical regulators of specific oxidative stress and antioxidative genes involved in the exacerbation of AMD pathogenesis.

## 4. Discussion

Our laboratory recently reported that *P. gingivalis* invades and survives within retinal epithelial cells (RPE), the main target of AMD, for a protracted period of time in vitro [20]. Here, we aimed to investigate the physiological association in vivo and the precise role of periodontal pathogens in retinal pathologies, specifically AMD. Choroidal neovascularization (CNV) is the foremost cause of vision loss while the most common cause of CNV is macular degeneration. Although, neovascular AMD (nAMD) affects only a lesser proportion (10–15%) of patients with AMD, it accounts for nearly 90% of severe vision loss. The usage of antivascular endothelial growth-factor (anti-VEGF) agents have been the gold-standard management for nAMD but with limitations in terms of treatment burden to the patients [89]. Due to an obscure multidimensional etiology and asymptomatic progression, management of AMD remains a challenge. Hence, a methodical understanding of the etiological mechanisms involved in the onset and progression of AMD is now a major requisite in this regard.

Our primary focus of this study was to examine whether *Pg* or oral-biofilm infection impact pathological neovascularization and retinal degeneration in a pre-existing AMD model. In studying the pathogenesis of macular degeneration, the laser-induced CNV mouse models are most widely employed to explore pathogenic angiogenesis in nAMD. Laser-induced models [71,78,79,90] are deemed as valuable tools in proof-of-concept experiments and successfully induced in rodents [91,92] and various nonhuman primates [93,94]. Laser-induced rupture of Bruch’s membrane and subsequent angiogenesis in the choriocapillaris is useful for modeling the features of vascular lesions in nAMD. Similarly, the ligature-enhanced PD model in rodents provides promising disease correlates [72,95]. However, a simple reproducible model that recapitulates the combined characteristics of both AMD and PD is presently unavailable. Therefore, we established, for the first time, an experimentally induced novel murine model representing both AMD+PD human diseases. Here, we employed the Li-CNV (as pre-existing AMD) + ligature-enhanced periodontitis (PD) model to analyze the vascular alterations in terms of CNV and leakage. We initiated and utilized this blended model to explore the link between AMD and PD in a physiological setting and to assess the disease comorbidity and resolution effects. The limitations of this model are attributed to the maintenance and integrity of the ligature in the oral cavity of mice throughout the experimental period. Nonetheless, this pioneering in vivo model is expected to be a good asset not only in understanding the molecular aspects of AMD, but also other ocular diseases.

The aggregated data from our current study highlights that chronic mono- or polymicrobial infection by the dysbiotic periodontal pathogens aggravate AMD phenotypes, specifically reduction of retinal thickness, subretinal and cuticular drusenoid lesions, sustained CNV, angiogenesis, and activation of inflammatory mediators. The analysis of vascular leakage in AMD+PD mice retinae by FFA demonstrated prominent blood leakage in both ligature-induced *P. gingivalis* (alone) and biofilm-infected Li-CNV retinae compared to the controls, markedly within a short duration of one week. It is interesting to note that there were large hyperfluorescent progressive vascular lesions persistent at 6 weeks, as visualized by FFA, which insist that chronic exposure to the oral dysbiotic pathogens will exacerbate pathological angiogenesis in the choroidal and retinal area. Other studies have reported the occurrence of hard exudate or drusen-like deposits in laser-induced mice models [77]. The type, size, and composition are useful biomarkers for analyzing the disease progression. This observation is coherent with our results, but strikingly, both *Pg*- and biofilm-infected AMD+PD retinae demonstrated progressively enlarged laser burn with extensive distribution of drusen-like deposits at 6 weeks of consistent exposure. Interestingly, this study found that up until 3 weeks, no phenotype was evident, but unpredictable rudiments of drusenoid deposits were initiated at the 4th week which gradually increased at 6 weeks in the subretina of AMD+PD mice. This suggests that *Pg* adapts to the retinal environment and alters retinal homeostasis culminating in perpetual retinal tissue damage. Evident from OCT imaging is an apparent reduction in the retinal thickness at the lesioned spots initially marked at the end of first week, which progressed uninterrupted at 6 weeks of time. These data altogether support the notion that oral pathogens can mediate the vascular alterations leading to initiation of drusen deposits, increased vascular permeability and angiogenesis in the CNV-lesioned retinae in a chronic condition.

The noninvasive imaging procedures further confirmed the disruption of Bruch’s membrane at multiple regions of the AMD+PD-infected mice retinae. The immune-privileged status of the retina is compromised by the disordered integrity of the Bruch’s membrane, an important site of drusen genesis, and the outer retinal barrier. We speculated that the compromised Bruch’s membrane and propagating angiogenesis in the choriocapillaris facilitate further entry of periodontopathogens and exacerbate the underlying neovascular changes in the AMD retinae. These results from our study denote that *Pg*/biofilm infections sustain laser injury and perpetuate CNV, a hallmark of advanced wet AMD. In a similar context, chronic systemic inflammation instigated by PD is correlated with neuroinflammation seen in Alzheimer’s disease (AD), where *Pg* has been detected in the brain of postmortem samples from AD patients [96], invading the brain tissue through compromised blood–brain barrier [97,98,99,100,101]. Interestingly, in the orally infected (*Pg* and biofilm) mice retinae, we detected significant expression of *Pg* and *Pg*-fimbrial 16S-rRNA genes which are critical in colonization and binding to other biofilm species. *Pg* was detected more than 1.5-fold in both *P. gingivalis* (monobacterial)- and biofilm (polybacterial)-infected mice retinae. This suggests the intriguing possibility that *Pg* disseminated through systemic circulation, adapted physiologically and genetically to the in vivo choroidal/retinal environment, where they altered immune homeostasis and key inflammatory mediators of AMD [28]. However, further study is warranted to demonstrate the host–pathogen interaction and the fimbrial ligand–receptor binding activities of *P. gingivalis* in different retinal cell types.

Several studies have noted that specific inflammatory and angiogenic factors were significantly up-regulated in AMD eyes [80]. Remarkably, our present analysis of inflammatory mediators relevant to both PD and AMD in AMD+PD mice retinae revealed significant (*p* < 0.01) up-regulation of pro-inflammatory [Tnf-α (≥6 folds), Tgf-β1 (≥7 folds), Il-1β (≥3 folds)] and decreased expression of anti-inflammatory (Il-10; 0.8-fold) markers consistent with advanced AMD. TNF-α is an important target molecule in the treatment of CNV in mice as it promotes CNV by up-regulating the production of Vascular endothelial growth factor (VEGF) through reactive oxygen species (ROS)-dependent β-catenin activation [102,103]. TNF initiate the angiogenic environment of active CNV lesions by reducing the levels of bone morphogenetic protein-4, which are the members of TGF-β super family [104]. Besides, TGF-β plays a vital role in the formation and development of CNV through Smad2/3-VEGF/TNF-α signaling pathway in wet AMD [105]. Similarly, IL-1β is a pro-angiogenic factor which stimulates VEGF, the most essential factor involved in both physiological and pathological neovascularization. Likewise, IL-10, a multifunctional molecule, may act as an anti-inflammatory and may be reported in reduction of angiogenesis, RPE cell apoptosis and in limiting cell proliferation and migration, by regulating the expression of VEGF [106]. In our study, IL-10 is significantly down-regulated, which could be attributed to vasculogenesis and progression of CNV in AMD+PD mice retinae, possibly by activation of VEGF. In wet AMD, the injured RPE cells have been found with augmented expression of IL-8 in addition to VEGF and monocyte chemoattractant protein-1 which attract monocytes from the choriocapillaris resulting in the breakdown of the blood–retina barrier and angiogenesis [107]. The activation of STAT3 by IL-6 receptor, a key mediator in subretinal fibrosis, promotes CNV and the levels of IL-6 is correlated with the size and activity of CNV in AMD patients [108,109]. Studies showed that dysbiosis of gut leads to increased intestinal permeability and a chronic low-grade inflammation characteristic of inflammaging, with elevated levels of IL-6, IL-1β, TNF-α, and VEGF-A leading to exacerbation of pathological angiogenesis [108,110,111]. Moreover, oxidative stress may also up-regulate VEGF expression in the retina and induce CNV. It is noteworthy that VEGF is shown to be elevated in patients with PD [112]. Interestingly, our results show that the vasculogenic factor VEGF and the angiogenic factors IL-6 and IL-8 were significantly increased more than 4- and 3-fold, respectively. Furthermore, we previously noted that *P. gingivalis* promotes immunosuppression and angiogenesis through a two-hit receptor–ligand process involving DC-SIGN^+hi^/CXCR4^+hi^ and IDO^+hi^/FOXP3^+hi^-driven pathway [39]. The current data shows significantly increased Foxp3 expression in the AMD+PD mice retinae, underscoring that PD incite an immunosuppressive state in the AMD retinae [113]. This data is highly consistent with other findings and signifies that PD could be a key promoter of the exacerbation of AMD pathology.

Intriguingly, a growing body of evidence highlights the strong association of inflammation and elevated oxidative stress (OS) in PD [114,115,116]. The pathogens in the inflamed periodontal pocket trigger the release of reactive oxygen species (ROS), namely hydrogen peroxide and superoxide by activation of the polymorphonuclear neutrophils and macrophages, which is crucial for the survival of anaerobic bacteria such as *P. gingivalis* [117,118]. Similarly, multitudes of investigations have highlighted the central role of oxidative damage in the retina contributing to inflammation and CNV, observed in AMD phenotype [119]. However, there is no evidence to associate this link in the etiopathogenesis of these chronic inflammatory diseases of our interest. Hence, we postulated that experimental PD may exacerbate AMD through OS and investigated a specific group of OS and antioxidant genes in AMD+PD mice retinae. Interestingly, in the retinae of lasered ligature-induced mice, *Pg* and biofilm markedly up-regulated many OS-related genes including Perk, Atf6 and Bip, whereas it down-regulated antioxidative genes specifically Nrf2, Ho-1, Gclc, Gclm, Gpx1, Sod1, and Prdx1, which are elemental in AMD pathophysiology. Recently, several studies have indicated that in AMD, elevated cellular stress in the RPEs can induce prolonged stress in endoplasmic reticulum (ER), which in turn triggers an excessive expression of VEGF and consequently CNV. This remains a crucial step in the conversion from dry form of AMD to a wet type. An extensive crosstalk occurs between the mediators of oxidative stress and unfolded-protein stress; protein-kinase-like ER kinase (Perk), activating transcription factor-6 (Atf6) transducers in the regulation of VEGF expression, which is highly significant in CNV formation and eliciting AMD-related pathological changes [84,88,120,121]. Moreover, chronic overexpression of binding immunoglobulin protein (BiP) can trigger neovascularization [122]. Our study demonstrates significantly increased expression of Atf6 (>3 folds), Perk (>2 folds), and Bip (>1.5 folds) in the retinae of AMD+PD mice, thereby insisting contribution of PD in the etiology of oxidative stress-induced neovascularization.

As a cellular defense against inflammation and oxidative insults, the nuclear factor erythroid 2-related factor 2 (Nrf2) acts as a versatile transcription factor through an orchestrated expression of specific antioxidant genes [87,123]. Upon stimulation, Nrf-2 upregulates heme oxygenase-1 (Ho-1), Gclc and Gclm subunits, which are the antioxidant response elements target genes [124,125,126]. Under oxidative stress, the expression of Nrf2 mRNA was impaired in the RPE of aged mice than in younger mice [127,128] and amplified the susceptibility to oxidative stress in the RPE [129,130], while Nrf-2 knockout mice have been reported to develop conditions analogous to AMD [46]. In addition, Nrf-2 regulates the production of glutathione in the RPE, through Gclc and Gclm [131], and renders protection against photooxidation. Nrf-2 also plays a protective role in the periodontal tissues by enhancing cytoprotective effects as it decreases the inflammatory signaling and oxidative damage in tissues [132]. Notably, our results showed (≤ 3-fold) reduced expression levels of Nrf-2, Gclc, and Gclm in the retinae of AMD+PD mice, while Ho-1 is more than 8-fold significantly (*p* < 0.01) down-regulated. Glutathione peroxidase-1 (Gpx-1) is a gatekeeper in counteracting ROS and a major intracellular antioxidant enzyme playing important roles in AMD [47] and is shown to exacerbate retinal neovascularization in mice [47,133]. The levels of Gpx have also been correlated with the activities of periodontal pathogens and the progression of PD [134]. Similarly, superoxide dismutase (SOD) is an important component of the endogenous antioxidant defense system in the retina, playing a significant role in protecting the photoreceptors and RPE cells from oxidative damage [116,135,136]. We have previously demonstrated that VEGF-B treatment up-regulated Gpx1, Sod1, and Prdx1 and down-regulated many oxidative stress genes in the retinae of rd1 mice [47]. Here we found Gpx1, Sod1, and Prdx1 were significantly (≤−2 folds) down-regulated in the PD+AMD mice retinae. These findings imply that PD confers a role in the up-regulation of important oxidative stress and dysregulation of antioxidative genes, involved in exacerbation of neovascularization and AMD pathogenesis. It is striking that although *Pg* alone in low-grade numbers provides a consistent activation of the above-mentioned biomolecules, it expresses a synergistic effect in the polymicrobial consortium in a combined inoculum. With this background, it is important to underscore the pathogenic role of periodontopathogens and oral dysbiosis that it induces, in instigating chronic aberrant retinal inflammation, as in AMD.

## 5. Conclusions

Age, chronic inflammation, and common life-style risk factors could be the bridging elements in the pathophysiology of PD and AMD, thereby advocating a justifiable correlation between these inflammaging diseases. Given this, we comprehend that oral dysbiosis through periodontal infection could be a persistent source of low-grade inflammation and pathological angiogenesis in AMD. Collectively, the data from this current study denotes that persistent insult with key periodontal pathogens and chronic over-activation leads to exacerbation of retinal vascular events through stimulation or regulation of inflammatory, angiogenic, oxidative stress, and antioxidant molecules, and may contribute to the pathophysiology of AMD. The present data will advance our existing knowledge of the etiopathology of AMD, while denoting that counteracting adverse inflammatory effects and oxidative stress by prevention of PD will be an effective therapeutic target for management of this vision-threatening disease.

## Data Availability

The datasets generated and analyzed during the current study are available from the corresponding authors on request.

## Supplementary Materials

The following are available online at https://www.mdpi.com/2076-3921/10/2/309/s1, Table S1: List of mouse primers for SYBR Green-qPCR analysis; Figure S1: Oral pathogens augmented vascular leakage in AMD+PD mice retina; Figure S2: Advancement of AMD pathogenesis in AMD+PD mice retinae; Figure S3: Reduced retinal thickness and infiltration of immune cells in the CNV area and choriocapillaris of AMD+PD mice; Figure S4: Intensified neovasculogenesis in the choroidal and retinal area of AMD+PD mice.
