# Supplementary material for: Exacerbation of AMD Phenotype in Lasered CNV Murine Model by Dysbiotic Oral Pathogens
Source: Antioxidants (Basel). 2021 Feb 18;10(2):309. doi: 10.3390/antiox10020309 (PMC7922506; doi:10.3390/antiox10020309)
Supplement: Supplementary file 1 [file antioxidants-10-00309-s001.pdf]

Supplementary materials

Exacerbation of AMD phenotype in Lasered CNV Murine Model by Dysbiotic Oral Pathogens

Pachiappan Arjunan[1,4*], Radhika Swaminathan[1], Jessie Yuan[1], Mohamed Elashiry[1], Amany Tawfik[2,4], Mohamed Al-Shabrawey[2,4], Pamela M Martin[3,4], Thangaraju Muthusamy[3], and Christopher W. Cutler[1]

[1]Department of Periodontics, Dental College of Georgia, Augusta, GA 30912, USA; parjunan@augusta.edu; rswaminathan@augusta.edu; jeyuan@augusta.edu; moelashiry@augusta.edu; chcutler@augusta.edu
[2]Department of Oral Biology and Diagnostic Sciences, Augusta, GA 30912, USA; amtawfik@augusta.edu; malshabrawey@augusta.edu
[3]Department of Biochemistry & Molecular Biology, Augusta, GA 30912, USA; pmmartin@augusta.edu; mthangaraju@augusta.edu;
[4]Vision Discovery Institute, Augusta University, Augusta, GA 30912, USA.
* **Corresponding author**: PARJUNAN@augusta.edu

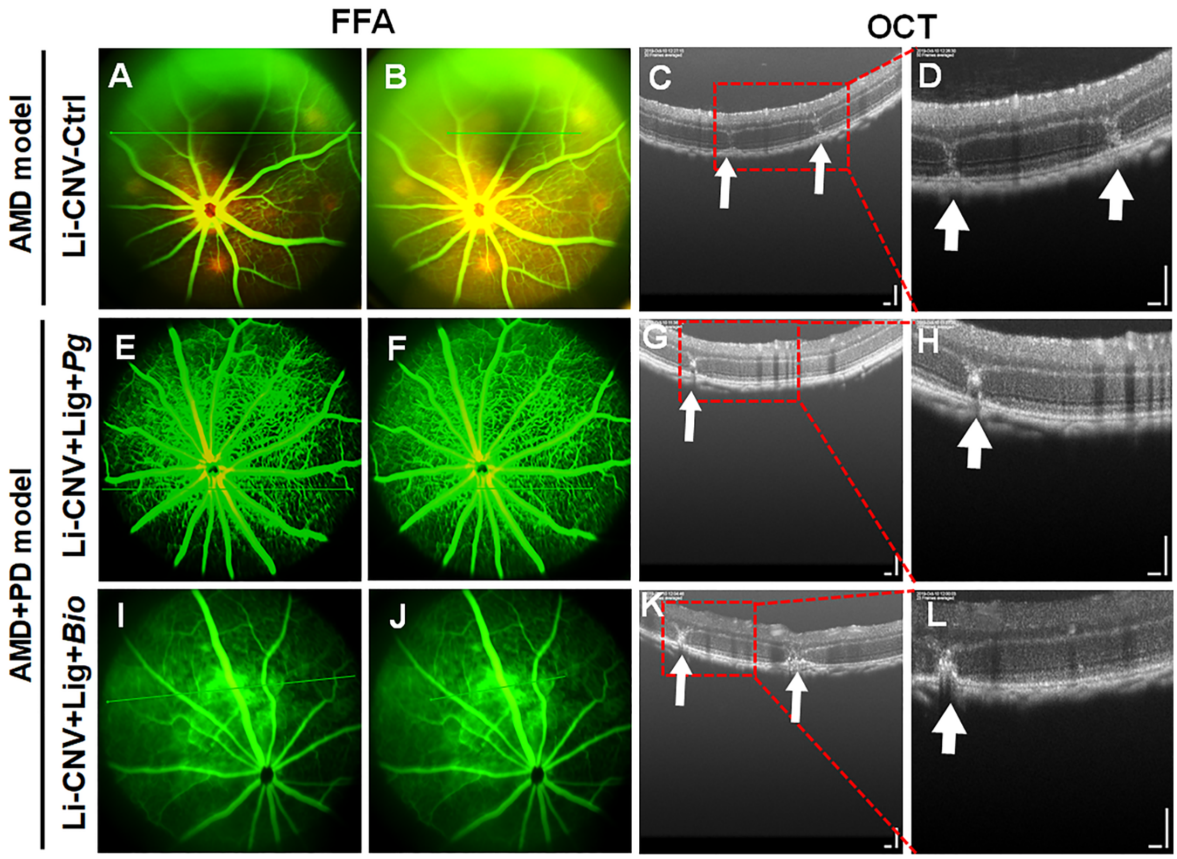

S. Figure 1. Oral pathogens augmented vascular leakage in AMD+PD mice retina.
A) After laser induction**,** mice were ligated with *Pg* or biofilm infection, AMD+PD murine model established as described in the methods. Fundus fluorescence Angiography (FFA) show vascular leakage in Li-CNV control (**A, B** with laser burn spots), Li-CNV+Ligature+*Pg* (**E, F;** shows more fibrovascular formation), and Li-CNV+Lig+Biofilm (**I, J**) after a week of laser and ligature induction; n=18. Spectral domain optical coherence tomography (SD-OCT) after 1 week of Li-CNV-Control (**C, D**), Li-CNV+Lig+*Pg* (**G, H**) and Li-CNV+Lig+Biofilm (**K, L**) infected mice retinae. OCT shows ruptured Bruch's membrane (BM, white arrows) induced by laser treatment in Li-CNV control (**C**), Li-CNV+Lig+*Pg* (G) and Li-CNV+Lig+Bio (**K**). Boxed areas in C, G and K show an enlarged region as D, H and L (with laser lesion, arrows), respectively.

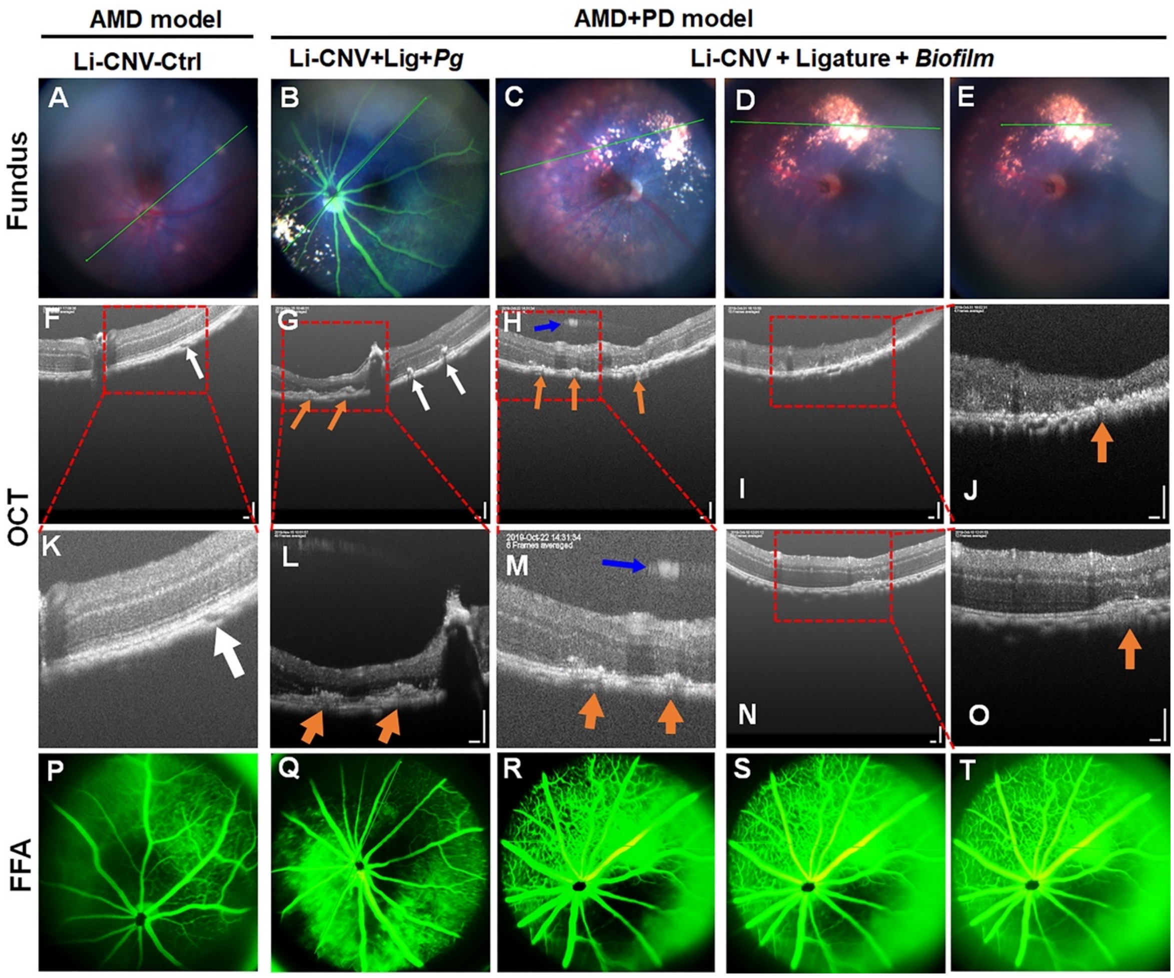

S. Figure 2. Advancement of AMD pathogenesis in AMD+PD mice retinae. Representative images of fundus (**A-E**), SD-OCT (**F-O**), and FFA (**P-T**) after 6 weeks of Li-CNV-Ctrl (**A,F,K,P**), Li-CNV+Lig+*Pg* (**B,G,L,Q**) and Li-CNV+Lig+Bio (**C-E, H-J, M-O, R-T**) infected mice retinae, respectively. **A-E**) Increased CNV lesion like morphology were observed by fundus imaging analysis in *Pg* (**B**) and biofilm (**C-E**) persistently infected retina compared with CNV-control (**A**). **F-O**) OCT images further demonstrates chronic CNV lesion (white arrows) in *Pg* (**E**) and Biofilm (**F**) after 6-weeks with vitreal (blue arrow) and subretinal angiogenesis compared to Li-CNV control (**F, K**). Orange arrows point out the drusenoid deposits located above the RPE layer. Boxed areas in F, G, H, I and N show an enlarged region as K, L, M, J and o, respectively. **P-T**) FFA displays increased blood leakage after 6 weeks compared to CNV.

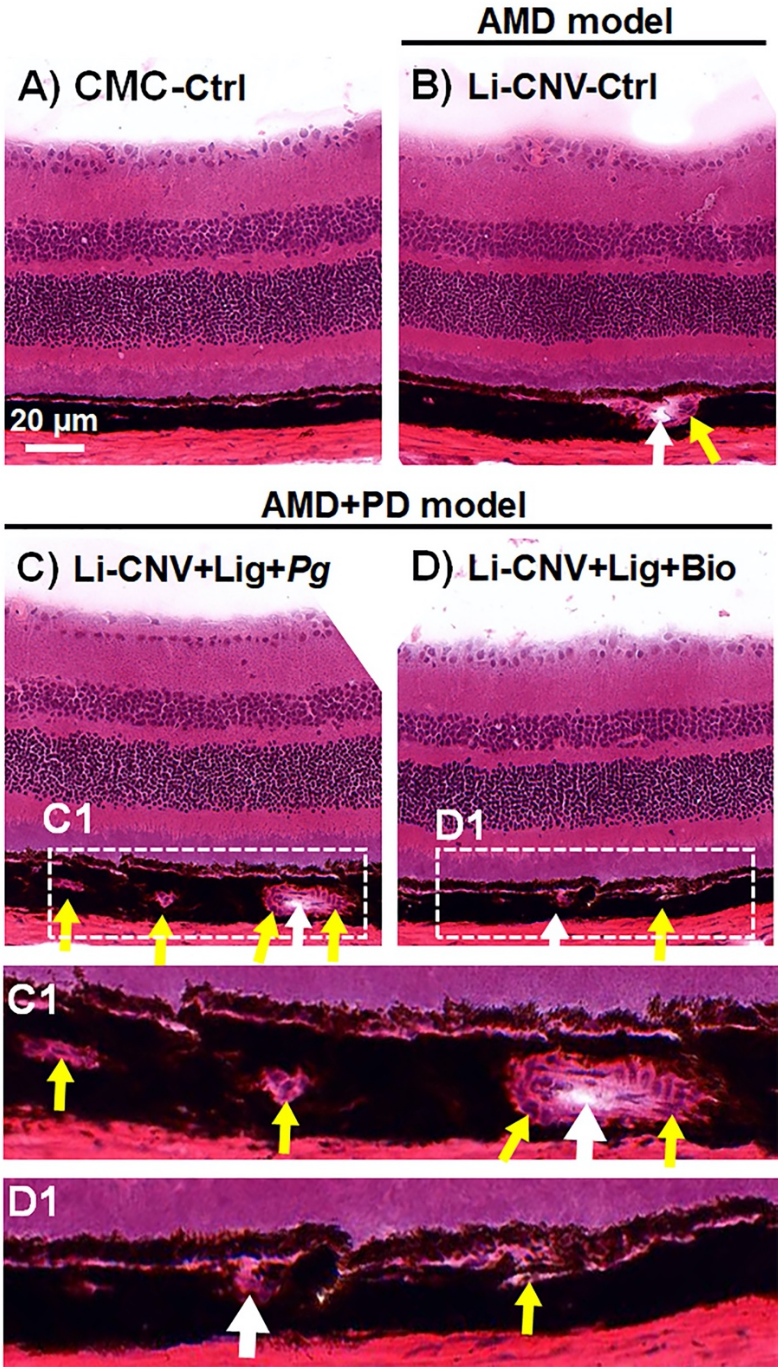

S. Figure 3. Reduced retinal thickness and infiltration of immune cells in the CNV area and choriocapillaris of AMD+PD mice. **A-D**) Representative images of hematoxylin and eosin staining illustrates thickness of retinal layers of Li-CNC+Lig+*Pg* and Li-CNV+Lig+Biofilm orally infected mice models after 6 weeks compared to CMC and Li-CNV controls. White arrows show the CNV areas. **B-D; C1 and D1)** White arrows indicate the CNV spots (empty space) while yellow arrows shows infiltration of immune cells in choriocapillaris of the AMD+PD retina. (Scale bar: 20 µm).

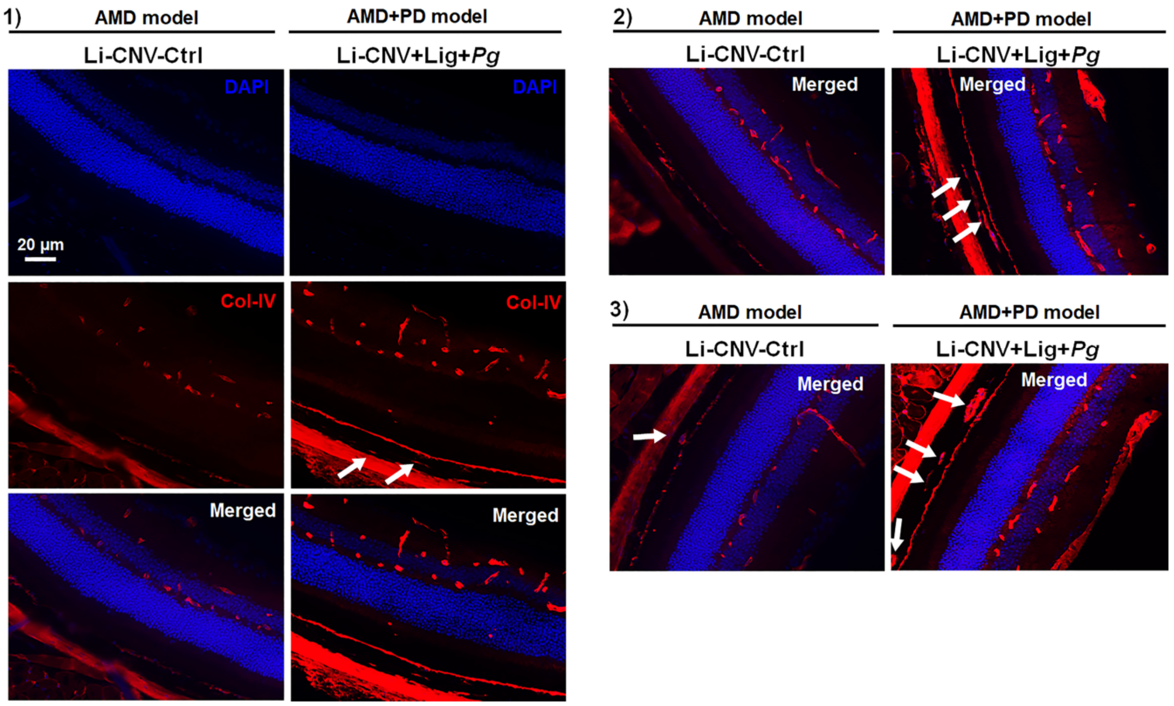

S. Figure 4. Intensified neovasculogenesis in the choroidal and retinal area of AMD+PD mice. 1-3) Representative images of immunofluorescence staining shows more Col-IV positivity in the AMD+PD mice retinas and choroidal region (white arrows) compared with CNV control. (Scale bar: 20 µm).

Table S1. List of mouse primers for SYBR Green-qPCR analysis

Gene Name	Description	Sense Strand (FW) (5'-3')	Anti-Sense (RV) (5'-3')
Vegf-A	Vascular endothelial growth factor A	aggctgctgtaacgatgaag	tgtcctatgtgctggctttg
Il-6	Interleukin 6	cacggccttccctacttcac	ctgcaagtgcatcgttgt
Il-8	Interleukin 8	gggtggtactgcgtatcctg	agacaaggacgacagcgaag

Gpx1	Glutathione peroxidase 1	gggactacaccgagatgaacg	ccgcaggaaggtaaagagc
Sod1	Superoxide dismutase 1	gtgtgcgtgctgaagggcg	cttcatttccacctttgccc
Prdx1	Peroxiredoxin 1	cacggagatcattgctttcag	ggtatcactgccaggtttcc
Nrf2	Nuclear factor erythroid 2-related factor 2	cgagatatacgcaggagaggtaaga	gctcgacaatgttctccagctt
Ho-1	Heme oxygenase 1	gatgacacctgaggtcaagca	cagctcctcaaacagctcaat
Gclc	Glutamate-cysteine ligase, catalytic subunit	aacacagacccaacccagag	ccgcatcttctggaaatgtt
Gclm	Glutamate-cysteine ligase, modifier subunit	tcgcctccgattgaagatgg	ttttacctgtgcccactga
